# Elucidating linear programs by neural encodings

**DOI:** 10.3389/frai.2025.1549085

**Published:** 2025-06-18

**Authors:** Florian Peter Busch, Matej Zečević, Kristian Kersting, Devendra Singh Dhami

**Affiliations:** ^1^Department of Computer Science, Technical University of Darmstadt, Darmstadt, Germany; ^2^Hessian Center for AI (hessian.AI), Darmstadt, Germany; ^3^Centre for Cognitive Science, Technical University of Darmstadt, Darmstadt, Germany; ^4^Foundations of Systems AI, German Center for Artificial Intelligence (DFKI), Darmstadt, Germany; ^5^Department of Mathematics and Computer Science, Eindhoven University of Technology, Eindhoven, Netherlands

**Keywords:** XAI, linear programming, attributions, neural encodings, machine learning

## Abstract

Linear Programs (LPs) are one of the major building blocks of AI and have championed recent strides in differentiable optimizers for learning systems. While efficient solvers exist for even high-dimensional LPs, explaining their solutions has not received much attention yet, as explainable artificial intelligence (XAI) has mostly focused on deep learning models. LPs are mostly considered white-box and thus assumed simple to explain, but we argue that they are not easy to understand in terms of relationships between inputs and outputs. To mitigate this rather non-explainability of LPs we show how to adapt attribution methods by encoding LPs in a neural fashion. The encoding functions consider aspects such as the feasibility of the decision space, the cost attached to each input, and the distance to special points of interest. Using a variety of LPs, including a very large-scale LP with 10k dimensions, we demonstrate the usefulness of explanation methods using our neural LP encodings, although the attribution methods Saliency and LIME are indistinguishable for low perturbation levels. In essence, we demonstrate that LPs can and should be explained, which can be achieved by representing an LP as a neural network.

## 1 Introduction

With the rise in popularity of Deep Learning in recent years which was corroborated by its tremendous success in various applications (Krizhevsky et al., 2012; Mnih et al., 2013; Vaswani et al., 2017), the popularity of methods which help to understand such models has increased as well (Sundararajan et al., 2017; Selvaraju et al., 2017; Hesse et al., 2021). The latter works constitute a new sub-field within artificial intelligence (AI) research, often referred to as explainable artificial intelligence (XAI). While XAI has tried a wide variety of methods and techniques to unravel the “black-box” of deep learning models, many restrictions can be found regarding the notion of explainability or interpretability that is expected and sought (Stammer et al., 2021). Therefore, to move beyond simple “heat-map” type of attributions, explainable interactive learning research [XIL; see for instance Teso and Kersting (2019)] poses one such alternative. However, in our work, we explore alternative models rather than other data streams or alternative definitions, moving “beyond” conventional attribution methods, i.e., we consider Linear Programs (LPs). While there has been considerable progress in increasing the understanding of neural networks (NNs), the complexity of deep models has received significant attention from the field of XAI, even though other fields might benefit from such techniques as well—for example, LPs from mathematical optimization.

Interestingly, LPs are, at first glance, on the opposite spectrum of model complexity, and they give the impression of being “white-box”. Representing problems with linear constraints and relationships, LPs can be useful and, in fact, are used for a multitude of real-world problems, and there exist many solvers capable of solving even high-dimensional LPs. It is even possible to use, solve, and backpropagate through LPs in a NN (Paulus et al., 2021; Ferber et al., 2020) and recent research has even shown the potential of using NNs to help solve LPs (Wu and Lisser, 2023; Li et al., 2024).

**Figure 1:**
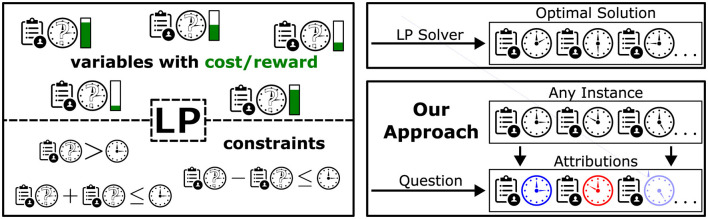
Task assignment example. The LP sketched here consists of variables describing the amount of time spent by a person on a specific task. Constraints restrict the space of feasible assignment plans. An LP solver can calculate the optimal assignment plan with respect to some cost/reward (**top right**). We propose to also look at attributions of assignments to get additional insights into the role of the individual assignment values (**bottom right**).

While a solver can be used to calculate the optimal solution of an LP, some applications could benefit from an increased understandability of their underlying LPs. To better illustrate how our method aims to go beyond optimal solutions, consider a scenario as shown in Figure 1. A company wants its employees to be able to perform multiple tasks instead of only working on a single problem. The company's overall goal is to assign the amount of time each person spends on each task, i.e., a variable represents a person-task pair, see top left in Figure 1. In other words, a solution consists of a full assignment plan with information on how long each person is supposed to work on each task. There are several constraints. Each person only has a specific amount of time available. For each task, there is a minimum amount of time required to finish the necessary work and a maximum amount of time that should not be exceeded. There could be further constraints like a minimum and maximum amount of time spent on each task per person or even person-specific constraints where certain individuals are not allowed to work on a specific task. Even with such constraints, there can be multiple solutions. In order to prefer a feasible solution over another, a score can be assigned to each variable where, if possible, more time should be assigned to variables with higher scores (green bars). This score could represent how much a person would like to work on the respective task, and the optimal solution would represent the assignment plan where the overall happiness is maximized without violating any constraints. This entire task assignment problem can be formulated as an LP. Given a large number of tasks, persons, and constraints, one can see how such a problem might quickly become quite complex.

Now, conventionally, one can use an LP solver on this problem, resulting in an optimal solution (top right of Figure 1). We propose to, in addition, get further insights into the role of the individual task assignments in an assignment plan (i.e., variable setting or instance) under consideration of the constraints. Different possibilities to do so can be seen as different “questions” to be asked on which the attributions for the respective assignment plan will depend.

**Figure 2:**
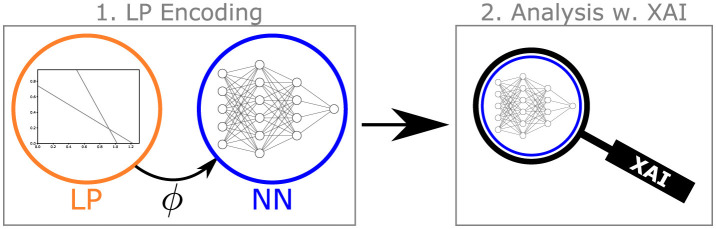
Schematized overview. For the first time, we apply XAI to LPs. To justify said application, we first propose an encoding ϕ of the initial LP, which is then used for learning a NN. Subsequently, said NN is analyzed using XAI (best viewed in color).

To achieve this goal, we propose to combine the field of XAI and LPs by utilizing the methods from XAI to increase the understanding of LPs. Figure 2 illustrates our basic approach. Given an LP, we use an encoding function ϕ to create data based on that LP, which is then used to train a NN. In the second step, we use common XAI methods mostly designed for use in Deep Learning to analyze the so-trained NN. By introducing different encodings for an LP, we create meaningful learnable tasks for the NN models. This, in turn, allows for applying any attribution method designed to help explain NNs, and hence, we can make use of the XAI literature to gain a better understanding of the given LP. We look at the attribution methods Integrated Gradients (Sundararajan et al., 2017), Saliency (Simonyan et al., 2014), Feature Permutation (Breiman, 2001), and LIME (Ribeiro et al., 2016).

Other attribution methods exist that are based on similar principles (e.g., perturbations, gradients) or have different goals, such as evaluating the importance of single layers within a NN (Dhamdhere et al., 2019; Shrikumar et al., 2018). We focus on the aforementioned attribution methods as they are well-known methods and based on different principles.

Getting closer to the goal of making LPs “properly explainable”, such that this refers to an intuitive, human-level understanding, would have incredible implications for science and industry (well beyond AI research). Scheduling problems (Jaumard et al., 1998; Garaix et al., 2018) could be looked at from an additional angle, considering aspects outside of the optimal solution. Energy systems researchers could design sustainable infrastructure to cover long-term energy demand (Schaber et al., 2012). Also, LP explainability naturally comes with strong implications within AI research, for instance in *quantifying uncertainty and probabilistic reasoning* (Weiss et al., 2007).

Overall, we make the following contributions: (1) We introduce an encoding function that distinguishes feasible from infeasible instances, including the cost of LP solutions, two functions focusing on the constraints, and a function using the LP vertices. (2) We look at the attribution methods and compare them depending on these encodings. (3) We once again explain the importance of selecting an appropriate baseline for Integrated Gradients. (4) We show similarities between Saliency and LIME, and we propose the property of *Directedness* as the main discriminative criterion between Saliency and LIME on the one hand and our Feature Permutation approach on the other hand.

We make our code publicly available at: https://github.com/olfub/XLP.

## 2 Background and related work

First, a short introduction to linear programs is given. After that, the necessary background on XAI is explained, including short descriptions of the attribution methods Integrated Gradients, Saliency, Feature Permutation, and LIME. This section ends with a list of general properties of attribution methods.

### 2.1 Optimization using linear programs

A linear program (LP) consists of a cost vector **c** ∈ ℝ^*n*^ and a number of inequality constraints specified by **A** ∈ ℝ^*m*×*n*^ and **b** ∈ ℝ^*m*^. The objective of an LP is to find a point (or instance) **x** ∈ ℝ^*n*^ which minimizes the cost **c**^*T*^**x**, is non-negative (**x**≥**0**), and does not violate the constraints specified by **A** and **b** in the form of **Ax** ≤ **b**.

There can either be no solution, one solution, or infinitely many solutions (Hoffman et al., 1953). The minimization task is the objective function and can also be written as a maximization task of the negative cost (i.e., *maximizing the gain* instead of *minimizing the cost*). In this paper, unless stated otherwise, when talking about the *constraints*, we refer to **Ax** ≤ **b** and not **x**≥**0**. When referring to a certain number of constraints in a specific LP, we refer to such a number of rows in **A** and corresponding elements **b**.

Sensitivity analysis (Bazaraa et al., 2008; Ward and Wendell, 1990; Saltelli and Annoni, 2010) on the LP and its dual can be used for explaining LPs. For example, it can answer questions on the impact of changes of some value in **c**, **A**, or **b** on the optimal solution or even of adding an entirely new constraint to the LP. These approaches, as well as infeasibility analysis (i.e., how an unsolvable LP can be changed to have at least one feasible solution) (Murty et al., 2000), can result in very useful insights on an LP for a specific question. Overall, our approach is inherently different from sensitivity analysis. While there are explanations that are easiest to obtain using sensitivity analysis, our approach of utilizing various types of encoding functions and attribution methods introduces entirely new possibilities for explaining LPs.^1^

### 2.2 Attribution methods from XAI

We follow the standard notion defined in Sundararajan et al. (2017):

Definition 1. Let *F*:ℝ^*n*^ → ℝ be a NN and $\text{x}=\left(x_{1},\ldots ,x_{n}\right)\in {\mathbb{R}}^{n}$ denote an input. Then we call ${\text{A}}_{F}\left(\text{x}\right)=\left(a_{1},\ldots ,a_{n}\right)\in {\mathbb{R}}^{n}$ attribution where *a*_*i*_ is the contribution of *x*_*i*_ for prediction *F*(**x**).

Therefore, the attribution **A**_*F*_(**x**) for the instance **x** consists of one attribution value for each feature of **x**. Such an attribution value *a*_*i*_ for the *i*-th feature describes the contribution of that feature to the output. How exactly this contribution should be understood depends on the attribution method.

The attribution methods here were chosen with the aim of enabling a comparison of different approaches. “Captum” was used to apply these methods (Kokhlikyan et al., 2020). We briefly cover the attribution methods relevant to this work's analysis. For a more general overview of XAI methods, surveys exist that extensively cover the entire breadth of this field (Schwalbe and Finzel, 2023; Abhishek and Kamath, 2022).

#### 2.2.1 Integrated gradients

Sundararajan et al. (2017) proposed an attribution method for deep networks that calculates attribution relative to a baseline. Formally, we are given ${\text{IG}}_{i}\left(\text{x}\right)=\left(x_{i}-x_{i}^{\prime }\right)\times \overset{1}{\underset{\alpha =0}{\int }}\frac{\partial F\left(\text{x}\prime +\alpha \times \left(\text{x}-\text{x}\prime \right)\right)}{\partial x_{i}}d\alpha$ where *x*_*i*_ is the *i*^*th*^ element of **x** and **x**′ is the baseline.

#### 2.2.2 Saliency

Simonyan et al. (2014) proposed a straightforward approach in which the attribution is obtained by taking the predictive derivative with respect to the input. Therefore ${\text{SAL}}_{i}\left(\text{x}\right)=\frac{dF}{dx_{i}}\left(\text{x}\right)$.

#### 2.2.3 Feature permutation

In the following, we consider a perturbation-based notion to feature permutation to allow for comparison in later sections. Feature Permutation (Breiman, 2001) requires multiple instances for calculating the attribution. The general idea is to use a batch of instances, iterate over every feature, permute the values of the respective feature in that batch, and then derive the attribution for each feature and instance by calculating the difference in predictive quality before and after the permutation. Since we want to have attributions for only single instances, we first define the *feature importance* (*FI*) function as acting on batches, that is $FI:X\subset {\mathbb{R}}^{m\times n}\to {\mathbb{R}}^{m\times n}\text{,}$ where X is the space of all batches of *m* instances with *n* features. *FI* takes a batch of instances and calculates attribution for each instance and feature by applying permutation. To obtain attribution for a single instance, we generate instances around the input instance using perturbation. We say that function *p*(**x**, *m*)∈ℝ^(*m*+1) × *n*^ generates *m*∈ℕ random perturbations of **x** and returns those perturbations and **x** (**x** in the last column). With *FI* and *p*, we can now apply the feature permutation algorithm of “Captum” on a single instance using *FI*°*p*, but we still need to decide how to use this attribution for this batch to obtain an attribution for just one instance. To this end, we generate one perturbed instance around the input point, calculate the attribution of this batch of two points using feature permutation **P** = (*FI*_*i*_°*p*)(**x**, 1) (where *FI*_*i*_ returns the feature importances of feature *i* only), and return only the attribution with respect to the original input instance. In order to decrease the impact of randomness, this is repeated several times, and the attributions are averaged. Therefore, this feature permutation approach FP is given by ${\text{FP}}_{i}\left(\text{x}\right)=\frac{\overset{10}{\underset{j=1}{\sum }}{\text{P}}_{:,2}}{10}\text{,}$ where **P**_:, 2_ is the second column of **P** (the column representing the attribution with respect to the original input instance).

#### 2.2.4 LIME

Ribeiro et al. (2016) used a strategy entirely different from the aforementioned attribution methods. The key idea of LIME is to train a surrogate model. While this model will only be able to reliably predict accurate results for the area around the input instance, this reduced complexity aims to make this additional model more *interpretable*. We have LIME_*i*_(**x**) = **w**_*IM*_ where **w**_*IM*_ are the weights of the linear interpretable model surrounding **x**. Then we simply state LIME_*i*_(**x**) = *R*(*p*(**x**), *F*_*B*_(*p*(**x**))) where *p*:ℝ^*n*^ → ℝ^*j*×*n*^ is a function which generates a batch of perturbed data $X_{P}\in {\mathbb{R}}^{j\times n}$, *F*_*B*_ is a batch-version of *F* ($F_{B}:{\mathbb{R}}^{j\times n}\to {\mathbb{R}}^{j}$, *F*_*B*_(**X**_*P*_) = (*F*(*x*_1_), …, *F*(*x*_*j*_))) and *R* is a ridge regression model ($R\left({\text{X}}_{P},F_{B}\left({\text{X}}_{P}\right)\right)={\text{argmin}}_{w}||F_{B}\left({\text{X}}_{P}\right)-{\text{X}}_{P}w|{|}_{2}^{2}+||w|{|}_{2}^{2}$).

#### 2.2.5 General properties of attribution methods

There are several well-known properties of attribution methods that will be useful later. An attribution method is *(a) Gradient Based*, if the attribution method relies on calculating gradients (Ancona et al., 2019). It is *(b) Perturbation Based*, if the attribution method uses perturbations to generate data around the input point (Zeiler and Fergus, 2014). *(c) Completeness* is satisfied if the attribution method relies on a baseline **x**′, and $\overset{n}{\underset{i=1}{\sum }}A_{F}\left(\text{x}\right)=F\left(\text{x}\right)-F\left(\text{x}\prime \right)$ is true (Sundararajan et al., 2017). And we refer to *(d) Randomness*, if randomness is involved in the calculation for the attribution. The impact of randomness can be reduced at the cost of calculation time by increasing the number of samples, steps, etc.

## 3 Encoding priors for linear programs

So far, most XAI methods have focused on neural methodologies (Gunning and Aha, 2019). To justify the usage of XAI for LPs, we consider their similarities with neural models. Specifically, we first show how to encode LPs in a “neural” manner as this will enable the application of attribution methods on these LPs. One of the most obvious approaches here could be to use **c**, **A**, and **b** as inputs for the NN and the optimal solution x* for the output. This approach would give us attribution for the inputs, so **c**, **A**, and **b**. While we believe that such attributions would also carry valuable information, in this paper, we mostly focus on the information we can obtain from looking at one specific, single LP, where we consider **c**, **A**, and **b** fixed.

If we only train with a constant **c**, **A**, and **b** as inputs, the NN will simply learn to output the optimal solution x* without actual “learning”. Therefore, we need a task from which we can construct a dataset with different inputs and corresponding outputs. We do so by inputting instance vectors from the LP (**x** ∈ ℝ^*n*^). Now, we can define the output and, thus, the corresponding learning problem. This enables us to consider different aspects of an LP. We refer to such a learning problem as an *encoding* (ϕ) of the original LP. These encodings allow for the application of attribution methods from XAI on the LP in such a way that different kinds of insights can be gained depending on the choice of encoding function and attribution method. They should, therefore, be considered with the application of attribution methods in mind, as this is where their value comes from. Note that this approach requires a new NN to be trained for each encoding and LP, thereby learning the relevant properties of the respective encoding well enough so that XAI methods can be applied correctly. Obtaining enough useful data and finding good NN hyperparameters can be challenging. The following paragraphs cover various reasonable encodings that we initially propose and then systematically investigate.

### 3.1 Feasibility encoding

In a straightforward manner, we can distinguish between *feasible* instances, i.e., instances that do not violate any constraints, and *infeasible* instances, i.e., instances that violate at least one constraint. Using binary coding, we have $\phi (x)=\{\begin{array}{ll} 1 & \left(Ax\leq b\right)\\ 0 & (Ax>b). \end{array}.$. Note that for our LP encoding, we will usually keep the LP constraints **A**, **b** constant. Therefore, in our notion, the encoding function only depends on the optimization variable **x**.

### 3.2 Gain-penalty encoding

In this approach, the feasible instances get assigned their corresponding gain and the score of an infeasible instance now depends on how much it violates the constraints. This is done by finding an ϵ-close (ideally the closest) feasible point, calculating the gain of that point, and reducing it, depending on the distance between these two points. Consequently, infeasible instances with only a small constraint violation have almost the same gain as the closest feasible instances, but infeasible instances with large violations get assigned increasingly smaller scores (gains). To some degree, real-world applications might justify this approach since the constraints are not completely prohibitive but rather because their violations are simply too costly to be efficient. The Gain-Penalty encoding is defined as $\phi (x)=\{\begin{array}{ll} c^{T}x & \left(Ax\leq b\right)\\ c^{T}x_{f}\left(1-\min\left(1,\frac{||x-x_{f}|{|}_{2}}{||x_{f}|{|}_{2}}\right)\right) & (Ax>b)\text{,} \end{array}$ where **x**_*f*_ = argmin_**x**_*i*__||**x**_*i*_−**x**||_2_ is the closest feasible instance in the setting where **c**, **A**, **b**, >**0**. Naturally, there exist various sensible variations to this formulation.

### 3.3 Boundary distance encoding

This encoding considers the boundary between feasible and infeasible instances. By calculating the minimum of **b**−**Ax**, we can find out if an instance lies on the boundary and obtain a score indicating how large the biggest violation is or, for feasible instances, how much space there is until the closest constraint would be violated: ϕ(**x**) = min(**b**−**Ax**). By taking the absolute of this encoding |ϕ(**x**)| (Absolute Boundary Distance encoding), we can treat the output as a distance to the boundary (*margin*), changing the behavior of attribution methods.

### 3.4 Vertex distance encoding

Here, the distance to the nearest vertex is used. A vertex of an LP is any feasible point lying on either the intersection of two constraints or on the intersection of a constraint with an axis (or at the origin). Note that there always exists an optimal solution on one of those vertices, but since this approach does not include the cost vector **c**, it does not contain any information about which of those vertices is an optimal solution. Overall, the Vertex Distance encoding is defined as $\phi \left(\text{x}\right)=\underset{\text{x}}{\min}V$, with $V=\left\{||\text{x}-{\text{x}}_{v}|{|}_{2}|{\text{x}}_{v}\in {\text{X}}_{v}\right\}$ where **X**_*v*_ denotes the set of vertices.

Why should one use a NN to learn encodings when this requires training an entirely new model? Generally speaking, it is possible that this NN does not learn the encoding without errors. However, there is one important difference compared to standard NN applications. In our approach, we always know all the true inputs and outputs. Therefore, we can not only easily evaluate whether the model in general managed to learn the problem well, but also specifically check how accurate the model's prediction is for specific inputs whose attributions we compute. So while training some encodings on large-scale problems can still be challenging and time-consuming, the risk of unknowingly getting “bad” attributions is relatively low. On the other hand, using a NN offers many new possibilities. As a universal function approximator, a NN can learn all the introduced encodings (and more), even if they were to contain other constraints or elements that could not be formulated as a LP. Not only does this make it possible to focus on different aspects of the LP, but it also means that any attribution method from XAI can be applied. Depending on the choice of encoding and attribution method, this leads to different attributions and, thus, insights.

## 4 Properties of LP encodings and attribution methods

Having introduced the main methodology used in this paper, we now consider several properties of encodings and attribution methods and discuss the implications of using NNs to learn LPs.

### 4.1 Properties of linear program encodings

Upon establishing various sensible encodings ϕ, we now propose reasonable key properties for the discussed ϕ that we can return to when discussing how attribution methods behave on different encodings. To ease notation later on, we define the set $X\subset {\mathbb{R}}_{\geq 0}^{n}$ such that the properties are only related to instances that are not infeasible for every single LP by construction (since **x**≥**0**). We propose the following key properties.

#### 4.1.1 Continuity

We call an LP encoding ϕ continuous if $\forall \text{x},\text{k}\in X.\left(\underset{\text{x}\to \text{k}}{\lim}\phi \left(\text{x}\right)=\phi \left(\text{k}\right)\right).$

#### 4.1.2 Distinguish class/distinguish boundary

If an LP encoding ϕ computes values in such a way that the feasibility of the point can be inferred from its output, we say that ϕ satisfies Distinguish Class. Formally, ∃f,∀x∈X.(Ax≤b↔f(ϕ(x))). Similarly, Distinguish Boundary is satisfied if we can infer whether a point lies on the decision boundary: ∃g,∀x∈X.(Ax=b↔g(ϕ(x))).

#### 4.1.3 Boundary extrema

This property is concerned with whether the boundaries of the LP are at the extrema of ϕ. There are multiple possibilities for defining such a property. We postulate that a method satisfies Boundary Extrema if an extremum (maximum or minimum) of ϕ lies on the decision boundary and if this extremum does not appear outside the boundary. The extremum may appear more than once on the boundary itself. Let *P*_*i*_: = min(**Ax**_*i*_−**b**) = **0**, then formally we have that there $\exists {\text{x}}_{i},\forall {\text{x}}_{j},{\text{x}}_{k}\in X$ such that *P*_*i*_∧((ϕ(**x**_*i*_)>ϕ(**x**_*j*_)∨*P*_*j*_)∨(ϕ(**x**_*i*_) < ϕ(**x**_*k*_)∨*P*_*k*_)).

### 4.2 Properties of attribution methods

Previously, we stated some well-known properties of attribution methods. In addition, we now propose a new concept as well as another property for attribution methods, which will be useful for discussing differences of attribution methods afterward.

#### 4.2.1 Neighborhoodness

Being more of a concept rather than a property, Neighborhoodness describes how large a region around the input point is considered for the attribution. For example, Neighborhoodness of perturbation-based approaches usually depends on the perturbation's “strength”.

#### 4.2.2 Directedness

We say that Directedness is satisfied if the attribution for a feature indicates how increasing that feature would change the output. For example, attribution would be positive if increasing that feature increases the output and negative if increasing that feature decreases the output. This increase might be local or over a larger interval, therefore the property does not directly depend on the Neighborhoodness of the attribution method.

Table 1 gives an overview of the previously introduced properties of our LP encodings and how the considered attribution methods fall into properties such as Neighborhoodness, Directedness, and other established properties.

**Table 1:** Properties of encodings and attribution methods.

	**F**	**G**	**B**	**A**	**V**
Continuity	×	✓	✓	✓	✓
DistinguishClass	✓	×	✓	×	×
DistinguishBoundary	×	×	✓	✓	×
BoundaryExtrema	×	✓	×	✓	✓
		**IG**	**S**	**FP**	**L**
GradientBased		✓	✓	×	×
PerturbationBased		×	×	✓	✓
Completeness		✓	×	×	×
Randomness		×	×	✓	✓
Neighborhoodness(*)		×	✓✓	✓	✓
Directedness		×	✓	×	✓

On the left are properties of encodings; on the right are properties of attribution methods. F, Feasibility; G, Gain-Penalty; B, Boundary Distance; A, Absolute Boundary Distance; V, Vertex Distance; S, SAL; L, LIME. Regarding Neighborhoodness (*)Not binary, and thus requires alternate interpretation. We argue that IG generally has the least Neighborhoodness because the path from baseline to input can be very long. Saliency always considers a smaller area than the perturbation-based approaches FP and LIME.

### 4.3 Using neural networks for linear programs

One might question the benefits of bringing NNs into the area of mathematical optimization since learning NNs that accurately represent the underlying problem can be challenging. On the other hand, NNs are very powerful universal function approximators, which, given a good choice of hyperparameters and large amounts of data, can solve difficult high-dimensional problems. On the other hand, they notoriously lack guarantees for making correct predictions and can easily overfit the training data, thereby failing to make correct predictions outside of the training regime. In our experiments, we always train a model using a train set and evaluate it on a separate test set, thereby ensuring that our evaluation accurately reflects the model behavior in general and not just on the training data. When training our models, we discovered that overfitting is generally very small. This can be explained by the Continuity property of our encodings, which, if satisfied, says that the encoding of a datapoint that lies between two other datapoints should also lie between the encoding of these other two datapoints. Thus, a function correctly representing the underlying problem should be considerably easier to learn than one that strongly overfits the training data. Even for the feasibility encoding where Continuity does not hold, only the decision boundary is not continuous. Importantly, we can also easily obtain both the true value and the model prediction for any input. Therefore, one can easily assess whether the model prediction is accurate before inspecting the attributions. For our experimental evaluation detailed in the next section, our trained models learn the underlying encodings very well (for model errors, see the Supplementary material).

## 5 Empirical illustration

We empirically analyze the encodings, properties, and implications from the previous three sections using an illustrative 2-dimensional LP. Afterward, we show results on a large-scale LP and present a slightly different experiment where not all constraints are fixed (ParamLP).

### 5.1 Experimental setup

To compare different encodings, we consider an LP that allows for 2d-visualization of its polytope and use a feed-forward NN to train a model for each encoding introduced in Section 3. Another experiment only trains a NN for the Feasibility encoding but uses an LP with five dimensions. We then consider a very large-scale problem generated using FRaGenLP (Sokolinsky and Sokolinskaya, 2021) to investigate whether our methods still work for large LPs. Lastly, we present an experiment called ParamLP in which the constraint vector **b** is parametric and also passed into the NN along with **x**. For most experiments, we generate 100,000 random instances such that the complete LP polytope (set of feasible solutions) is covered and that about as many feasible and infeasible instances are being generated. For integrated gradients, we use the baseline **x**′ = **0** if not specified otherwise. In our experiments, FP and LIME use perturbations by generating instances around the input point up to a set maximum perturbation. We use a Ridge Regression model for LIME. In the Supplementary material, further extensive information on the problem and data generation and the experimental setup are presented.

### 5.2 Overview of results

**Figure 3:**
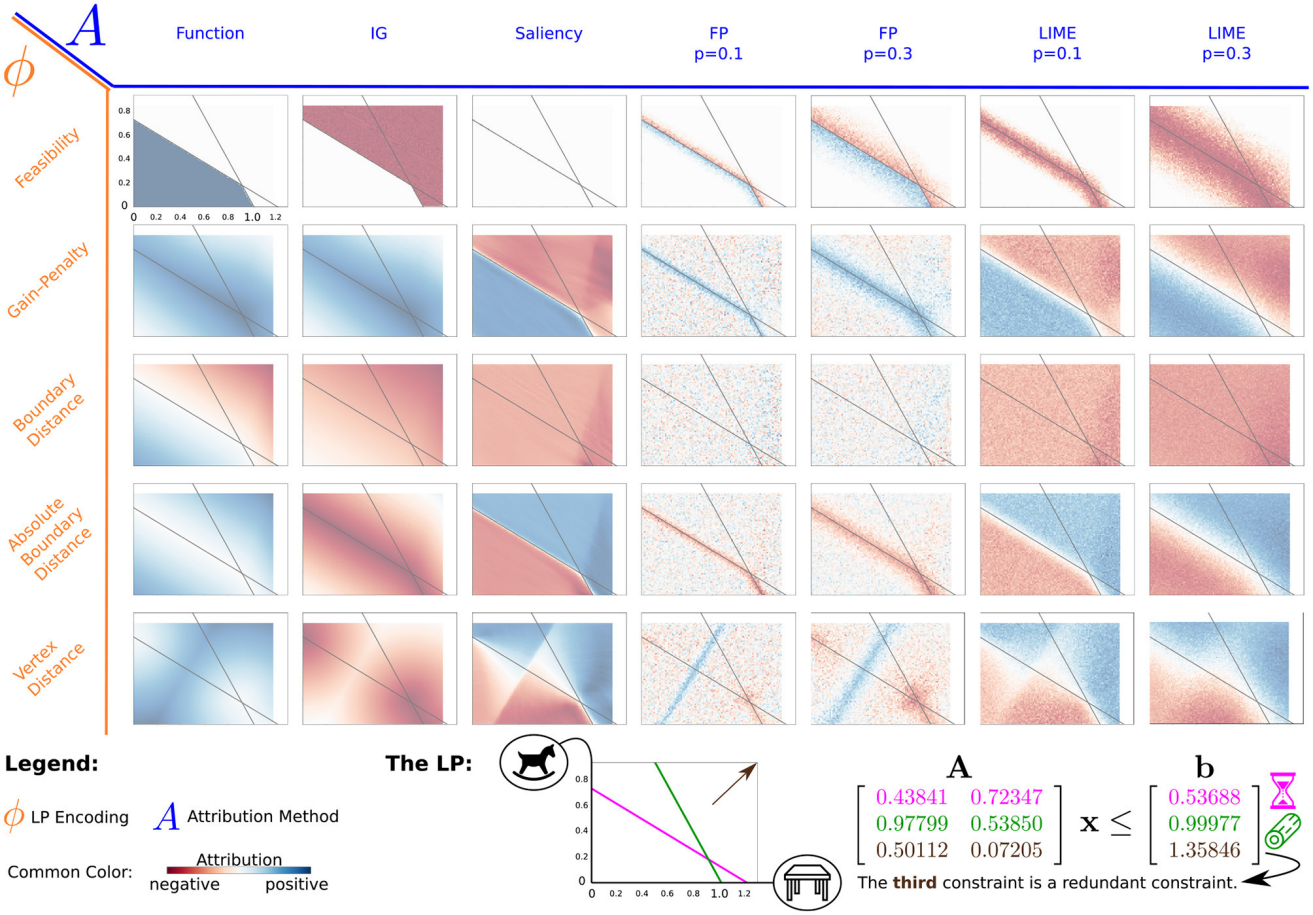
Overview-matrix of LP encodings and attribution methods. All plots show a single LP with two features, one horizontal and one vertical, where the gray lines indicate the constraints for that LP. This matrix of plots shows the summed-up attribution of both features (attribution sum). The encodings (orange, rows) are plotted against the different attribution methods (blue, columns). For FP and LIME, p indicates the maximum possible perturbation in any direction. The (rounded) numbers for the constraints of that LP are shown on the bottom right. The icons show an example application for this LP. A craftswoman could either work on a table or a wooden horse, but there are two relevant constraints that limit the amount of time and wood available. The amount of storage is a third constraint, which is irrelevant given the other two (best viewed in color).

In Figure 3, we show the encodings vs. attribution methods matrix (with summed-up feature attributions; refer to the Supplementary material for an example showing single feature attributions on the same LP). The only encoding using the cost vector (**c** = [0.5, 0.6]^*T*^) is the Gain-Penalty encoding. Here, the optimal solution lies at the intersection of the two constraints. We approximate the regions we generated data for with 100 × 73 pixels. Note that we ignored the vertex (0, 0) for the Vertex Distance encoding by including our prior knowledge that it cannot be the optimal solution. We refer to $\overset{n}{\underset{i=1}{\sum }}A_{F}\left(\text{x}\right)$ (i.e. the sum of the feature attributions for **x**) as the attribution sum. By analyzing these results, we aim to find out how encodings change the attributions of the respective methods. For the larger LPs and ParamLP, we look at how the Feasibility encoding in particular performs on higher dimensional examples.

78%^2^ for the large-scale (10,000-dimensional) LP. In the following, we focus on the most important insights derived from our experiments. Model errors as well as additional results and explanations can be found in the Supplementary material.

We use an example scenario for the 2-dimensional LP to illustrate possible applications. A craftswoman is working with wood. She can work on two projects, given by the axes in the LP. They are continuous, as their value can be seen as the progress made over the course of one day. Three constraints apply to her work. Firstly, there is only a limited amount of time. For example, the time available for one day is enough to finish one table and start working on a second one, but only about two-thirds of a wooden horse can be finished in that time (see the intersections of the purple time constraint with the two axes). Secondly, she only has a finite amount of material to work with, and the small wooden horse requires less material than the large table (green constraint). The third constraint depicts the storage requirements. Fortunately, the storage is big enough for this constraint not to matter, considering the other two constraints.

#### 5.2.1 Integrated gradients

The attribution sum for Integrated Gradients follows from its Completeness property: $\overset{n}{\underset{i=1}{\sum }}{\text{IG}}_{i}\left(\text{x}\right)=\phi \left(\text{x}\right)-\phi \left(\text{x}\prime \right)\text{.}$ Therefore, if Continuity, Distinguish Class, Distinguish Boundary, or Boundary Extrema is satisfied on ϕ, that same property also applies to IG. A benefit of Completeness is that the attribution for each feature indicates how large its contribution is relative to the change in output compared to the baseline output. In contrast to other methods with no such property, this makes it so that every attribution has a tangible meaning and that the attribution for a single instance can be understood without the need for other instances for comparison.^3^

If Completeness is satisfied, the important part of any attribution is how the contribution is divided amongst the individual features. For example, consider the craftswoman example with the Vertex Distance encoding in Figure 3. This encoding might be interesting for our craftswoman if the value (i.e., cost vector) for the table and horse is unknown and she simply wants to utilize time and material most efficiently but at the same time look for a configuration that could be optimal (i.e., a vertex). Here, the encoding scores can be seen as the amount of wasted work, either because of a constraint violation or simply because the assignment could be improved. The craftswoman uses a baseline of (0, 0) describing the smallest possible effort and looks at different points and their attributions. On the vertices, these attributions obtain the smallest value (far from 0), as here, the least amount of work is wasted. In addition, the attributions for the single features give information on how much the individual features are responsible for the change in output, i.e., the reduction of wasted work (also see the Supplementary material). For example, the vertices lying on the axis only get attribution on those features that changed compared to the baseline. This would be different if a baseline on the top right were chosen. Therefore, the main challenge when applying IG is choosing a sensible baseline. In our example, maybe using a vertex as the baseline could carry more meaning, as now the attributions would be compared to an “optimal” assignment where no work is wasted. However, the craftswoman would still have to decide which vertex to choose, as this also influences the resulting feature attributions.

Overall, applying IG on LP encodings can result in useful and understandable attributions, but those must always be interpreted with respect to the respective baseline. In addition, choosing an appropriate baseline is often not obvious.

#### 5.2.2 Saliency

Since Salience simply calculates the local gradient, its general behavior is easily explained. In accordance with satisfying Directedness, attribution for a feature is positive if the feature impact on the respective point is positive, negative if it is negative, and 0 otherwise. Notably, this also means that there is no attribution on local extrema (see encodings satisfying Boundary Extrema in Figure 3). Since Saliency has a very small Neighborhoodness, attribution usually requires some additional information to be useful. For example, attributions on and around a local maximum have different values, ranging from positive attribution, where the value increases toward the maximum, to 0 exactly on the maximum, and to negative attribution afterward. All those attributions are “correct”, but if you were to consider only one of those points and have no knowledge about the maximum there, you might draw false conclusions about the feature impact in that area. In this example, prior knowledge about that local maximum or at least considering multiple other points reduces that risk.

For example, consider the Gain-Penalty encoding for our craftswoman scenario. Up until the boundary, putting in more work increases the gain (value earned), but right after either the material or the time runs out, investing more is harmful. In addition, the single feature attributions also give information on how much a feature is worth increasing. Remember that such information can be far less obvious when working with higher dimensional LPs where constraints can be positive and negative, so when increasing a feature can also help satisfy a constraint.

One interaction worth discussing is how Saliency behaves on points where the encoding function is not differentiable. We can see this for the Absolute Boundary Distance encoding in Figure 3. Due to the absolute value function, points on the boundary are not differentiable. Still, the NN approximates this function in a differentiable way. Not only does this result in a 0 attribution (gradient) exactly on the boundary, but there is also a small area around the boundary where the gradient quickly, but not instantly, changes from 0 to the true gradient of the area around. Other non-differentiable points appear whenever an encoding does not satisfy Continuity. For the Feasibility encoding, this results in an extreme attribution very close to the boundary, where the NN approximates this discontinuity with a very steep function.

To sum up, attribution here reflects very local changes w.r.t. an increase of input features. Approximations of non-differentiable points by the NN influence Saliency attributions near those points.

#### 5.2.3 Feature permutation

Due to its perturbations, FP focuses on the output changes around the input point. Since it does not satisfy Directedness, attribution in areas with a steadily changing output in one direction can average to 0, as the positive change in one and the negative change in the opposite direction cancel each other out. If these two changes are equal, the remaining attribution results from the Randomness in the perturbation process.

Even though Directedness is not satisfied, there is a difference between negative and positive attribution, but it has to be understood differently. Because FP considers the area around it without consideration of direction, local minima (maxima) can be observed to have negative (positive) attribution because permuting features of instances around it increases (decreases) their function output.^4^ For example, suppose the craftswoman wants to assign to her apprentice how much he should work on the two tasks. She expects him not to listen perfectly and that he might work a bit more or less on either task. This is generally fine with her, but she does not want him to work in such a way that either takes too much time or wood. In other words, she expects some perturbations but does not want constraint violations. Using the Absolute Boundary Distance encoding, she can look at the attribution for an assignment that, on its own, is feasible, and if the chosen parameters filter out noise sufficiently, check whether the attribution is negative. Negative attribution is given if a random perturbation of the input plan, on average, decreases the distance to the boundary. In terms of the example, the apprentice would either use too few resources or require too many. On average, this is only bad if an assignment is close to the boundary, as here, the additional resources are not available, and the results of both kinds of perturbations are undesirable.

The degree of perturbation can be seen as a trade-off between precise, local results on the one hand and robust and emphasized attribution for larger areas on the other hand. All in all, attributions given by Feature Permutation consider any direction equally, resulting in a focus on local minima and maxima of the encoded function.

#### 5.2.4 LIME

In Figure 3, we can now observe how LIME with smaller perturbation (Neighborhoodness) looks increasingly similar to Saliency. This is just as true for the attributions of the features alone which indicate how a feature impacts the output with respect to an increase of that feature (Directedness). Unlike with Saliency, however, we can choose how large of an area around the input point should be considered by LIME. So, while small perturbations behave like Saliency, larger perturbations can help focus on the more overarching characteristics and make the procedure more robust against local errors and bad approximations of the NN. On the other hand, small, local perturbations allow for a more precise attribution of the respective points. Based on our experiments, the nature of LPs, and our noiseless data generation, we argue that the usual downsides of very local perturbations might not be as significant.

Again, consider the example where the craftswoman wants to find out how working on either task is bad for the constraints of time and material. Using the Boundary Distance encoding, it can be seen that an increase of work on either task is harmful with respect to the constraints, it costs material and time. However, there are some task assignments on the right side (working on the table a lot) where this impact is even stronger (i.e., larger, dark red values). This makes sense, as for many other assignments, the time constraint is the more restrictive one but, as can be confirmed by inspecting the precise values of the constraint matrix **A**, the second constraint (material) is violated more quickly when working on tables than the time constraint.

On discontinuities, LIME, because of its Neighborhoodness, can have very large attributions. The attribution, i.e., the slope of the linear model, is steeper the smaller the perturbations. Overall, LIME attributions show the same characteristics as those generated by Saliency. However, LIME allows for changing the Neighborhoodness, making it possible to consider either very local changes or a larger area.

#### 5.2.5 Higher dimensional LP

**Figure 4:**
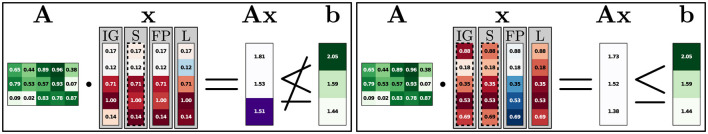
5-dimensional feasibility experiment. Here, an infeasible instance on the left and a feasible instance on the right are shown. In **A** and **b**, green indicates the element size. Constraint violations are marked purple in **Ax**. Attributions go from red (negative) to blue (positive). Attributions with dashed borders have very small absolute attribution values (< 0.01). All values are rounded.

We now consider the Feasibility encoding on a larger LP consisting of five feature dimensions and three constraints. Figure 4 shows one infeasible and one feasible instance with the corresponding constraints and constraint violations. Attributions are shown for all four attribution methods, with an individual color scale for each method and input. As before, *red* indicates negative, *white* zero, and *blue* positive attribution. The left instance in Figure 4 is infeasible as it violates the third constraint (1.51≰1.44). All attribution methods here focus on the last three features, which have the highest impact on the violation of that third constraint. For the feasible instance (right), the second feature has the least attribution. Here, both the second and the third constraints are somewhat close to violation, and while the last three features are important for the last constraint, the first feature is important for the second constraint. The Saliency (S) attributions and one IG attribution are marked with dotted lines indicating small attribution values (under 0.01). This results from the same type of behavior previously mentioned in Section 5.2.2. The discontinuous decision boundary is approximated by the NN in a continuous manner, resulting in instances close to the boundary to have outputs close to, but not exactly 0 or 1. This effect can even be useful, as the ratio of the feature attributions still contains information (without it, these attributions would all be 0).

#### 5.2.6 Large-scale LP

This large-scale experiment considers the Feasibility encoding for an LP with 10,000 features and 30 constraints. The model improves greatly upon the baseline of always predicting the most likely class, but many predictions are still false. The following figures show the 5 constraints with the largest violations (or closest to being violated) and the 5 features with the largest absolute attributions. IG uses the zero vector as its baseline. We did not find the results for FP or LIME very useful or interesting, and we leave the application of those on such large-scale LPs for future work.

#### 5.2.7 Integrated gradients

**Figure 5:**
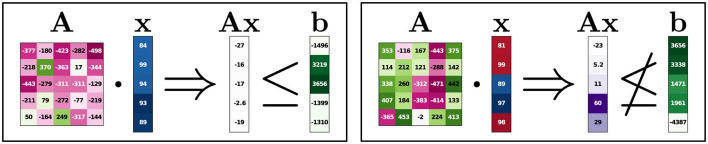
Large-scale Experiment, Integrated Gradients. Because of the large dimensionality of the LP, only selected columns and rows are shown. **Ax** was calculated using the full LP, before selecting the depicted rows. The values in **Ax** are in the order of 10^4^. The colors in **A** serve as an additional indicator of the elements (negative values are dark pink/purple, positive values are green). The predictions for the instances on the left and right are 0.82 and 0.53, respectively.

Since the LP used for this experiment has both positive and negative values in **A**, we can observe how features that help satisfy constraints have a high positive attribution (blue) if they correspond to large negative elements in **A** while those values that correspond to large positive elements in **A** have a high negative attribution (red). Furthermore, features for infeasible instances are strongly influenced by those rows in **A**, which are responsible for constraint violations, even if the prediction of the model is rather bad as shown in Figure 5. Note that, with the baseline being the zero vector, larger features tend to be assigned larger attributions, even if smaller features have the same relative impact. For example, if, compared to the baseline, one feature has double the impact per change by 1 compared to a second feature, the second feature still has a higher overall attribution if its value is twice as far away from the baseline. In some other instances, attributions do not seem to make as much sense. We assume the reason for this to be the errors in the model performance, although sometimes attributions seem useful despite poor model predictions.

#### 5.2.8 Saliency

**Figure 6:**
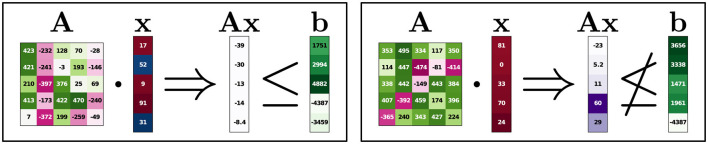
Large-scale experiment, saliency. Details as in Figure 5. The predictions for the instances on the left and right are 0.74 and 0.53, respectively.

Knowing that Saliency should, in theory, not have any attribution in the Feasibility encoding, it should still not be surprising that some attribution can be observed. Since the model is not perfect and learns a continuous function where a discontinuity should be, some attribution can be observed for many instances. The selected features presented in Figure 6 tend to correspond to columns that fit the type of attribution given. For high negative attributions (red), the columns tend to contain positive elements in **A**, and for high positive attributions (blue), the columns tend to contain negative elements in **b**. In other words, the attributions still show how the features impact the output, giving some useful information.

#### 5.2.9 ParamLP

**Figure 7:**
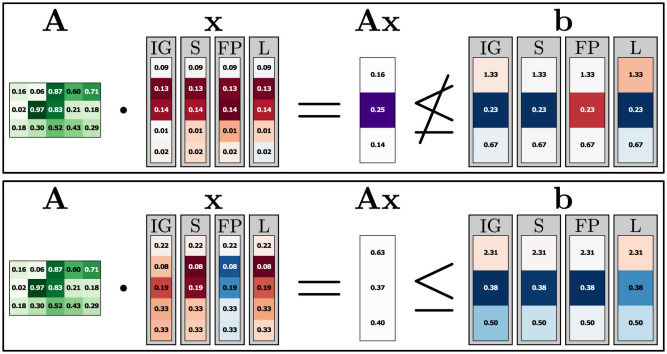
ParamLP experiment. Details as in Figure 5, with **b** now also being given into the NN as a parametric input. The predictions for the instances on the top and bottom are 0.00 and 0.98, respectively.

In this experiment, the NN gets both **x** and **b** as inputs and learns the Feasibility encoding using these inputs. For IG, we use the zero vector as a baseline for **x** and **0.1** as a baseline for **b** such that the baseline is an easily determined feasible instance (using **b** = **0**, the baseline would lie exactly at the decision boundary, possibly making attributions involving gradients less reliable). Following the same visualization procedure as for the higher dimensional experiments, we present the results of this ParamLP experiment in Figure 7. For **x**, the attributions for the infeasible sample again follow the same patterns as before. As for both the feasible and the infeasible sample, the second constraint is the one with the largest constraint violation (or closest to being violated), the attribution in **x** is mostly influenced by the second row in **A**. Here, the second and third elements have much larger values than the others; hence, the corresponding second and third elements in **x** obtain high attributions. Unsurprisingly, attributions on **b** for the top, infeasible instance clearly show that the violated constraint is the most important with respect to the model output (feasibility). For the feasible instance, the second constraint also gets most of the attribution since it is closest to being violated. In addition, the third constraint now has higher attribution values since it is also relatively close to **Ax**, which is not the case for the first constraint, where the attribution is closer to 0. Seeing how we can easily add inputs other than **x** into the NN to obtain other attributions, even on parameters of the LP itself, reaffirms the value of using XAI and NNs to help explain LPs and shows the versatility and potential of such an approach.

### 5.3 Relationships between attribution methods

Having examined the general behavior of the four attribution methods considered in this paper, we now go into detail about how they relate to each other. First, we argue that IG differs significantly from the other methods. While the “information” that IG uses to calculate its attribution consists of the path between baseline and input, all other three methods base their attribution on the area around the input. Looking at our results in Figure 3, we can see that especially Saliency and LIME show noticeable similarities, particularly if smaller perturbations are used for LIME.

#### 5.3.1 Similarity of saliency and LIME

The high-level description of how LIME functions is arguably similar to the underlying functioning of Saliency. Could this intuitive similarity on a high level hint toward more, possibly an equivalence under certain circumstances?

Conjecture 1. For a perturbation → zero, the attributions of LIME → Saliency.

**Figure 8:**
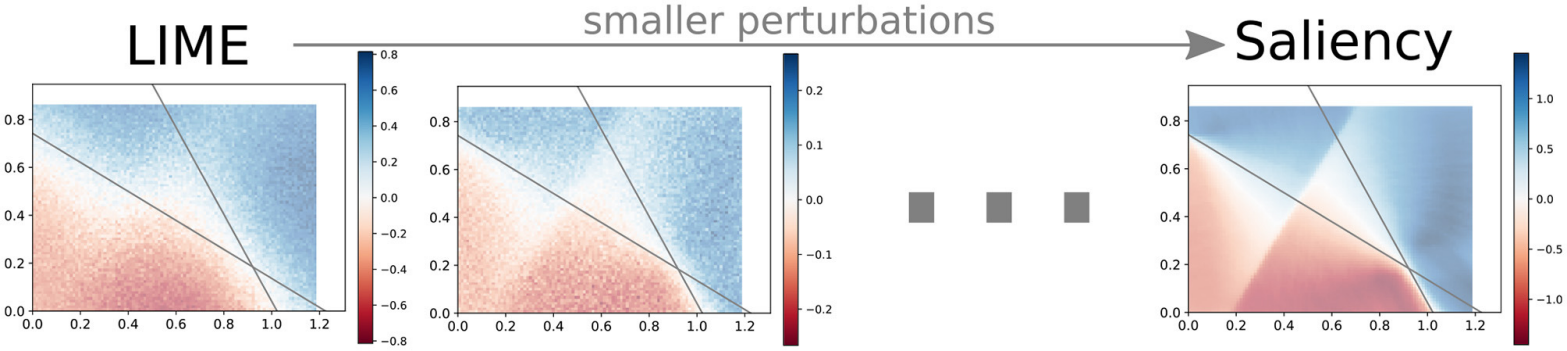
“LIME ≡ saliency”. With increasingly smaller perturbations, LIME comes visibly closer to Saliency. The decrease in attribution scores for smaller perturbations is due to the regularizer of the Ridge Regression model.

Both approaches calculate a value for a specific point with respect to how the output values around this point change, in other words, they both satisfy Directedness. So, if we perturb instances for LIME in such a way that they are infinitely close to the original input but not on the input itself (in which case there would be no attribution), then LIME approaches Saliency in the limit. We support this conjecture empirically (Figure 8). From that perspective, the main difference between LIME and Saliency is that the former allows for considering a larger area (perturbation function as a hyperparameter). However, even though the computation is different, we know from our experiments that FP and LIME use the same perturbations. So, if both those methods use the same “information” to calculate attributions, how can FP look different?

#### 5.3.2 How feature permutation differs from saliency and LIME

Just like Saliency and LIME, FP uses nearby regions to determine attributions. Since these methods base their attribution on similar information, could we again postulate a result on the triangular relationship? We conjecture on the approaches' similarity:

Conjecture 2. The main difference between Saliency and LIME on the one hand and FP on the other hand is Directedness. If you were to “*insert”* Directedness into FP, you would get an approach that behaves almost identically to LIME and, thereby, also to Saliency (for small perturbations).

Usually, FP does not consider “how”, i.e., in which direction a change happened, but only that it happened. For example, on a maximum, both Saliency and LIME (with small enough perturbations) would return little or no attribution because an increase leading up to the maximum is canceled out by a decrease going away from it.^5^ However, FP only considers that there is an average decrease in any direction if a feature is changed, so if that decrease is large, then that feature must be important. Using FP is not necessarily worse, and one might very well argue that it should be preferred in this example. But if FP would consider that *decreasing* the feature *decreases* the output and *increasing* the feature also *decreases* the output, it could use this information of direction (Directedness) to also return an attribution akin to Saliency and LIME.

**Figure 9:**
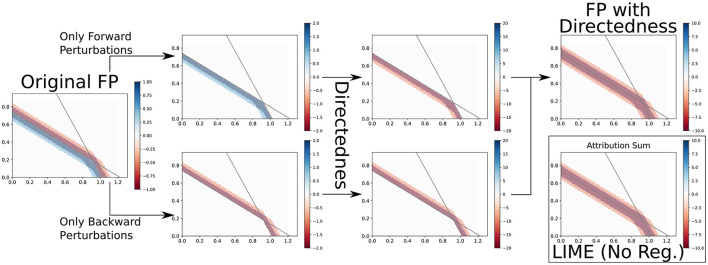
FP → LIME. If we make it so that FP distinguishes between forward and backward perturbations and scale attributions according to their distance from the input point, we can get results similar or even identical to LIME.

An example of the FP relation to LIME can be seen in Figure 9. This is a simplified example with only four perturbations, each of which only changes a single feature by 0.1. For this case, we even reach identical attributions, and while this is not generally true, the difference between LIME and FP after this transformation is also barely visible in most other cases. Also, note that we used Linear Regression instead of Ridge Regression to show equality more easily after the transformation.

## 6 Conclusion

We investigated the question of whether, and if so, how common attribution methods from XAI literature could be applied to settings beyond neural networks. Specifically, we looked at linear programs where we introduced various sensible neural encodings to represent the original LP. Various experiments were conducted to illustrate how several attribution methods could be applied and why the results are useful, even for very large and difficult linear programs. We show that LIME and Saliency have very similar results if small perturbations are used for LIME. By introducing the property of Directedness, we also found out how a perturbation-based Feature Attribution approach can be transformed to behave very similarly to LIME and, hence (for small perturbations), also to Saliency. We believe that the discriminative properties (Table 1) can guide the development of transparent and understandable attribution methods while also paving the road for more general applications in machine learning.

For future work, we can consider explaining special types of LPs as those used for quantifying uncertainty, such as in MAP inference (Weiss et al., 2007). The application to mixed-integer LPs or integer LPs such as Shortest Path or Linear Assignment might prove valuable. In addition, further investigating the scaling behavior of explanations generated for LPs or the cognitive aspects of whether and how LP attributions could be more “human understandable” seems critical.

## Data Availability

The datasets used in this study are fully synthetic and can be generated using the provided code. The repository can be accessed online via the following link: https://github.com/olfub/XLP.

## Footnotes

^1^For an in-depth comparison between sensitivity analysis and our approach, we refer to the Supplementary material.

^2^Only for this experiment, the baseline is 66.67% accuracy (otherwise 50%) because the number of infeasible instances is larger.

^3^Arguably, even IG can not explain instances individually, as every explanation requires a baseline reference.

^4^An increase in output leads to a negative attribution because the feature importance is calculated as the original output minus the new output (see https://captum.ai/api/feature_permutation.html).

^5^In both cases, the increase first and decrease later is w.r.t. an increase of the feature value, i.e., Directedness.
