# Supplementary material for: Elucidating linear programs by neural encodings
Source: Front Artif Intell. 2025 Jun 18;8:1549085. doi: 10.3389/frai.2025.1549085 (PMC12213677; doi:10.3389/frai.2025.1549085)
Supplement: Supplementary file 1 [file Data_Sheet_1.pdf]

Supplementary Material

1 SUPPLEMENTARY DATA

In this supplementary material, we first detail the problem and data generation for the LPs used in our experimental evaluation (Section 1.1). Afterward, we provide additional details on our experimental section by first detailing the experimental setup (Section 1.2) and then looking closely at additional experimental results that have not been discussed in depth in the main body (Section 1.3).

1.1 Linear Problem Generation

In order to properly investigate linear problems, we need to create data in a way that is somewhat representative, and that is not trivial. For all but the large-scale problem generation, we make a small simplification and only consider $\mathbf{b} \geq \mathbf{0}$, $\mathbf{A} \geq \mathbf{0}$, and $\mathbf{c} \geq \mathbf{0}$ such that all problems are solvable ($\mathbf{x} = \mathbf{0}$ is always feasible), the feasible regions expand into the positive space (all $\mathbf{x} \leq \mathbf{0}$ are infeasible) and the optimal solution is not trivial (larger $\mathbf{x}$ have higher gains then lower $\mathbf{x}$, hence, the optimal solution changes from problem to problem and is not at $\mathbf{0}$). We use different problem and data generation approaches depending on the linear problem size.

1.1.1 2-Dimensional Problem Generation

In two dimensions, we first randomly generate $\mathbf{A}$ and $\mathbf{b}$ in [0,1) and then multiply $\mathbf{b}$ by 2 (this is merely to increase the feasible space such that we do not have to deal with many decimal places for the feasible instances). Next, a balanced dataset of feasible and infeasible instances must be generated. To this end, the smallest axis intersections on both axes are determined and form a vector $\mathbf{x}_{\text{max}}$. Any instance larger than $\mathbf{x}_{\text{max}}$ must be infeasible, but not any instance smaller than $\mathbf{x}_{\text{max}}$ must be feasible. If we uniformly sample our dataset between the zero vector and $\mathbf{x}_{\text{max}}$, the ratio between feasible and infeasible instances will either be 50% (as is the case with only a single constraint) or larger than 50% (multiple different constraints). For this reason, we iteratively generate a dataset and then increase or decrease the max vector to generate more positive or negative instances until the class balance is deemed acceptable (between 40% and 60%). This dataset is then returned. Generally, our data generation generates a random $\mathbf{c}$ in [0, 1) but, for the purposes of this paper, we set it to $\mathbf{c} = [0.5, 0.6]^T$ such that the optimal solution does not lie on an axis, resulting in a more interesting problem for the Gain–Penalty Encoding.

1.1.2 5-Dimensional Problem Generation

When using the same approach as for the 2-dimensional problem, the hyperrectangle that is determined by the smallest axis intersections still contains all feasible instances. However, the larger the dimensionality, the larger the ratio of infeasible instances. For this reason, we employ a different data generation procedure for this problem. Here, we first randomly generate the constraint matrix $\mathbf{A}$ and the instances ($\mathbf{x}$). Then, we determine the probability under which a single constraint must be true such that the overall probability of all constraints being true is 50%. This can be calculated as $p_{\text{constraint}} = 0.5^{\frac{1}{m}}$, where m is the number of constraints, i.e., the dimensionality of $\mathbf{b}$. Now, we calculate $\mathbf{Ax}$ for all instances and set $\mathbf{b}$ as the $p_{\text{constraint}}$ quantile of the resulting vectors, thus ensuring that overall, roughly 50% of all instances is feasible and infeasible, respectively.

1.1.3 Large-Scale Problem Generation

We use FRaGenLP (Sokolinsky and Sokolinskaya, 2021), an algorithm for randomly generating high dimensional LPs, to generate an LP with 10,000 features and 30 constraints. In their algorithm, a hypercube of a specific size is defined, and the center of this hypercube is always feasible. We choose a hypercube size of 100, which means that the instance where all elements are 50 is feasible. FRaGenLP then generates random constraints in such a way that they cut into the hypercube. Using the resulting LP, only the dataset needs to be generated. To this end, we consider the hypercube of length 100. Due to the problem generation, most instances sampled uniformly in this hypercube are infeasible. To nevertheless obtain a relatively balanced dataset, our dataset is composed of three equally large parts. For the set of feasible and "easy" infeasible instances, we uniformly sample from the hypercube until the specified number of samples is reached (this is really fast for the infeasible part but requires much more time for sampling enough feasible instances since most other samples need to be discarded as infeasible). Since we now have a much less dense infeasible space, we generate a third set of instances as "difficult" infeasible instances. Here, the difference of an instance to the point we know is feasible (all features are 50) was decreased by 1% until the instance is feasible. Then, the data point representing the instance right before it becomes feasible is added to the dataset. The goal of this data generation procedure is to learn the decision boundary particularly well. However, note that this also results in the generation of many instances that are rather difficult to learn (because they are close to the decision boundary). This way, we have a baseline accuracy of 66.67% (always predicting infeasible), but accuracies above that are especially difficult to obtain since the set of feasible instances and the set of "difficult" infeasible instances are very close to each other. If a model learns to make correct predictions on most of the "easy" infeasible instances, it will be able to correctly predict most instances randomly sampled from the hypercube (as infeasible).

1.1.4 ParamLP Problem Generation

The ParamLP problem differs from the previous problem generations as now there is not one constant $\mathbf{b}$ but a different one for each sample $\mathbf{x}$. After randomly generating a constraint matrix $\mathbf{A}$, we first compute a reference vector $\mathbf{b}_{\text{ref}}$ as the sum over the constraints in $\mathbf{A}$. With $\mathbf{b}_{\text{ref}}$, the instance $\mathbf{x} = \mathbf{1}$ is feasible, but any larger instance is not. Using $\mathbf{b}_{\text{ref}}$, we again, similar to the two-dimensional problem, determine the smallest axis intersection to define the hyperrectangle for generating instances. In a calibration phase, we then generate data using this hyperrectangle and $\mathbf{b}_{\text{ref}}$ as the maximum values for $\mathbf{x}$ and $\mathbf{b}$, respectively, and iteratively adjust the size of the hyperrectangle until the percentage of feasible and infeasible instances within it is similar. The final dataset is then generated from scratch in the same manner but while keeping the hyperrectangle fixed.

1.2 Experimental Setup

In this section, we provide details on the experimental setup and training details for our experiments.

1.2.1 Model Architectures and Training Details

We use the same model architecture for all experiments except for the large-scale experiment. We here focus on all other experiments and give details on the large-scale experiment afterward in Section 1.2.2. The input dimension of our NN depends on the input size of the problem, i.e., the input size is the dimensionality of $\mathbf{x}$ (added with the dimensionality of $\mathbf{b}$ for the parametric LP experiment). This is followed up by 6 hidden layers with 4096 neurons each. After each hidden layer, a ReLU activation function is applied to the output. The output layer results are passed into a sigmoid function for the Feasibility encoding or returned directly for all other encodings. The size of the dataset always consists of 100k instances, of which half is

used for training and one quarter for test and validation each (although the validation set is only used for the large-scale problem). For training, batches of size 64 are used along with an Adadelta (Zeiler, 2012) optimizer, a starting learning rate of 1, and a learning rate decay of 0.7. The 2-dimensional problems are trained for 20 epochs, the 5-dimensional problem for 25 epochs, the parametric problem for 10 epochs, and the large-scale problem for 40 epochs.

1.2.2 Large-Scale Problem Hyperparameter Search

For the large-scale problem, we conducted a small hyperparameter search to find a network architecture where the model makes good predictions. To this end, we generated a dataset of 1M instances (half for training, one quarter for validation, and one quarter for testing) to try out different numbers of layers and neurons where the neuron count per layer either remains constant or the number of neurons gets halved for every consecutive layer. Additionally, we also tried out several batch sizes for training. In the end, we found out that a network with 11 layers with 8192 neurons each and a training batch size of 256 produces good results. We trained this model for 40 epochs. All the training information left unspecified in this subsection is identical to the other experiments. Even though a baseline accuracy of 66.67% can be reached by always predicting the infeasible class, our baseline accuracy of 81.78% is quite high as one-third of the instances were created to be very close to the decision boundary. Therefore, correctly classifying these and all feasible instances is extremely challenging; hence, our accuracy indicates overall decent model performance. Note that predictions on random instances from the considered space would be correct with an even higher percentage as most of these samples are further away from the decision boundary. We also found out that attribution, even of incorrectly classified samples, can provide useful information (e.g., a gradient can still be meaningful even if the associated prediction is a bit off). Further improvements with an even more optimized model might give even better results.

1.2.3 Technical Details

Our code is available at `https://github.com/olfub/XLP`. All models were built using Pytorch and trained on Nvidia DGX-clusters with A-100 40GB GPUs.

1.3 Additional Experimental Results

Section 1.3.1 shows the model errors for the models used in our experiments. After mostly looking at the attribution sums before, Section 1.3.2 considers attributions for single features. In Section 1.3.3, more information on the large-scale experiment is given.

1.3.1 Learning of the Encodings

In our experiments, the models learning the Feasibility encoding achieved the following accuracies on the test set: 99.98% (2-dimensional), 99.94% (5-dimensional), 99.73% (ParamLP), and 81.78% (large-scale experiment). The other encodings on the 2-dimensional problem had average errors (i.e., error per test sample) of 0.000057 (Gain–Penalty), 0.000004 (Boundary Distance), 0.000005 (Absolute Boundary Distance), and 0.000028 (Vertex Distance).

1.3.2 Single Feature Attributions

In Figure S1, feature attributions for both features on the example of the Vertex Distance encoding are shown. [1] These plots have been created using the same experiment as the corresponding one in the main paper. Generally, it can be seen how the methods also differ in their single feature attributions, and that

[1] We keep to the methodological details here and do not refer to the craftswoman scenario used in the main paper.

feature attributions can vary significantly depending on the respective feature. This section aims to inspect the results shown in Figure S1 to go into detail about the behavior of the attribution methods used in this paper and to explain the differences between the attributions for the two features.[2] For example, it can be seen how the negative attribution for the top left vertex when using IG results from the vertical feature, as this is the only feature different from the baseline $(0, 0)$. Compared to that vertex exactly, points further to the right even have some positive attribution on the horizontal feature, as this is responsible for increasing the score (here: distance from the nearest vertex). Since the overall distance is still smaller than at the baseline, these points can still have an overall negative attribution despite the positive attribution on one feature. How scores for single features differ can also be seen for the other attribution methods.

Integrated Gradients. Attribution for IG is obtained by calculating gradients on the path from a baseline to the input vector. As mentioned before, this experiment uses the origin as its baseline: $(0, 0)$. To illustrate this calculation, consider points that only differ from the baseline on the vertical feature (the far left of the shown plots). Here, the attribution for the horizontal feature remains at 0, as there is no change in this feature compared to the baseline, so it can not be responsible for any change in the output. Moving up towards the top left vertex, however, the vertical feature gets an increasingly larger negative attribution. Since the attributions of IG sum up to the difference in output between baseline and input instance (Completeness), this difference has to be reflected by the feature attributions. In this encoding, the function values encode the distance to the nearest vertex. The encoding at the baseline (origin) has some positive value, and the lowest values (the smallest possible distance, i.e., 0) can be found on any of the vertices. Therefore, the attribution for the vertical feature on the left vertex (where the horizontal feature remains unchanged compared to the baseline) must equal the negative difference between baseline output and 0, since otherwise, the Completeness property would be violated. Another interesting region in Figure S1 can be found on the top, roughly in the middle of our plot. Here is the same kind of negative attribution for the vertical feature but also some positive attribution for the horizontal feature. While the increased vertical feature decreases the distance to that vertex, the increased horizontal feature increases the distance, resulting in positive attribution for that feature. In other words, the horizontal feature being larger than its baseline value (0) has a positive (the distance increases) impact on the output. This same reasoning also applies to other regions but there, the attribution can be influenced by other vertices, resulting in different attributions. For example, being on the bottom right indicates a small distance to the nearest vertex there (one of the two vertices on the bottom right), which is why, for such instances, positive horizontal feature values can have negative attributions.

Saliency. Since Saliency uses the local gradient for its attributions, understanding gradients is mostly sufficient for understanding this attribution method. To put it simply, on a specific point, if a feature has a positive impact on the output, then the gradient is positive, and if a feature has a negative impact on the output, then the gradient is negative. This impact and therewith the gradient can also be 0. Let us consider the Saliency feature attributions in Figure S1. In accordance with Directedness, if increasing a feature would get the point closer to a vertex (decrease the distance), it gets negative attribution. If increasing a feature would get the point further away from a vertex (increase the distance), it gets positive attribution.[3] The white areas in this figure might benefit from some further explanation. First of all, there is (close to) 0 attribution on and around the vertices. The vertices are points where, in theory, the gradient should be undefined (because of the absolute value function used in the encoding). However, the NN approximates

[2] Readers which are very familiar with these methods might find large parts of the following explanations obvious, for less versed readers, these explanations hopefully can help to make the results more understandable.

[3] Keep in mind that, like with gradients, such statements of "increasing" and "decreasing" should not be interpreted with some specific amount of change but rather infinitesimal changes of the input.

the true underlying function in a continuous way, leading to a continuously changing gradient and an attribution of 0 on the vertices, which rather quickly reaches the "normal" gradient when moving away from those vertices in either direction. The white line starting at the bottom (somewhat left) and ending at the top (somewhat right) also has many points with (close to) 0 attribution because, in this region, the gradient switches signs. For the single feature attributions, there are also some white lines moving away from the vertices: to the top/bottom for the horizontal feature and to the left/right for the vertical feature. Those are areas where only that respective feature changes its gradient from one sign to another. For example, if a point is below the nearest vertex, then its vertical feature attribution is negative first and once the point is above the closest vertex, the attribution becomes positive. At some point in between, when the point is at the same height as the vertex, its feature attribution is 0. This is true both if these points are exactly under/above the vertex or slightly on one side. Combining this behavior for points on the left and right of the vertices results in a horizontal white line indicating no attribution next to the vertex. The explanation for the horizontal feature can be done the same way. Such white lines resulting from this type of behavior are not necessarily visible in the plot for the attribution sum since, here, the other features can have non-zero attributions. However, the attribution sum can also contain areas with no attribution even though there are single feature attributions because, in such situations, these get averaged out to 0 overall (this can also be seen in Figure S1).

Feature Permutation. The attributions of FP need to be interpreted differently as they are unlike the other attribution methods in Figure S1. For FP, the output of the input point is compared to the outputs of neighboring points (perturbations). If, on average, their output is larger, then the attribution of the input instance is negative, which describes the behavior seen around the vertices. If the neighboring points are, on average, smaller, then the attribution is positive. For the Vertex Distance encoding, this blue (positive) attribution can be observed on the line with an equal distance to both nearest vertices, as here, perturbing any feature in any direction creates a point closer to a vertex. Note that FP always considers only one feature changed at a time since perturbations are only created by perturbing a single feature. The single feature attributions also indicate how important the features are compared to each other. For example, on this aforementioned blue line, the attribution is larger for the horizontal feature. This makes sense, as moving to the left or right has a higher impact than moving up or down by the same distance.[4] The noise visible in many regions results from the randomness in the FP perturbations. For example, in many areas the score increases roughly the same in one direction as it decreases in the other direction, therefore the FP attribution should be around 0. However, if, due to randomness, perturbations are created more strongly in one direction, the average output is now predominantly influenced by that direction, resulting in an average positive or negative change and an attribution not close to 0. This can happen in either direction, which in Figure S1 results in noisy areas with many red and blue dots in an otherwise rather white region. Interestingly enough, the strength of this noise for both features here indicates in which areas which feature has a higher impact (this even compares to the respective Saliency feature attributions). The larger the perturbations (p in Figure S1), the broader the area considered for the attributions, resulting in less accurate (local) but increasingly robust attributions that represent more general changes.

LIME. The similarity between LIME and Saliency is not only present for the attribution sum but also for the single feature attributions. Instead of using the local gradient, LIME creates a small model for the input point based on perturbed instances around it. To summarize the resulting attributions briefly: Feature attribution for LIME is positive if larger (smaller) instances have larger (smaller) outputs, negative if larger

[4] Because the line is more vertical than horizontal, changing the horizontal feature can get a point closer to a vertex. In other words, since the vertical distance between the two relevant vertices is smaller than the horizontal distance, changing the horizontal distance here has a higher impact on the output.

(smaller) instances have smaller (larger) scores, and zero if either their outputs are smaller in one but equally larger in the other direction or if instances around the input have the same output as the input itself. Note that, as with Saliency, the direction of the change around the input instance matters (Directedness). Also, remember that, unlike FP, LIME uses perturbations for multiple features simultaneously. For larger perturbations, it can be seen that the attribution patterns become more blurred, and local details are disappearing. In some situations, this could possibly be an advantage and protect against noise, making the results more robust. There can be some slightly differing attributions of similar points due to the randomness in the perturbations.

1.3.3 Large-Scale Experiment

We provide a set of 10 examples from the evaluation of the large-scale experiment in Figures S2 (IG and Saliency) and S3 (FP and LIME). Feature Permutation and LIME use perturbations of up to 10. For IG and Saliency, the results in large parts fit the patterns also discussed for smaller LPs. For FP and LIME, the results are not so easy to understand. Here, the errors still left in the model might play an important role as there are many predictions made for the perturbations that could be false.

1.4 Comparison with Sensitivity Analysis

Sensitivity analysis (SA) (Bazaraa et al., 2008; Ward and Wendell, 1990; Saltelli and Annoni, 2010) is the process of analyzing the sensitivity of optimal solutions in linear programs. By calculating the dual of the linear problem, shadow prices can be determined, which provide information about the change of the optimal solution under changes of individual constraints (Gal, 1986). Generally, SA is useful for analyzing how small deviations in the constraints or cost function change the optimal cost and whether the optimal solution remains unchanged.

While our approach outlined in this paper overlaps with SA slightly, there are fundamental differences. We do not claim to strictly improve upon SA, but rather to introduce new ways of getting insights into linear programs beyond what SA offers. Both approaches have clear benefits. The main advantage of SA is that the entire analysis is based on strict, mathematical principles and, therefore, always calculates exactly what it is supposed to, without the need to train a NN model that encodes the desired behavior. In other words, the information that SA provides, such as shadow prices, can best be obtained using SA, and the strength of our approach lies not in improving upon SA when it comes to calculating just that. On the other hand, we introduce a broader approach for getting *different* kinds of insights into LPs by making use of encodings and attribution methods, generally moving beyond considerations about small perturbations and their effects on the optimal solution.

By choosing different encodings, we can consider aspects of the LP other than just the optimal solution. Many of our experiments focus on different choices of $\mathbf{x}$ and how their different features relate to the feasibility with respect to the set of constraints. However, we also include experiments where the cost function is considered (the Gain–Penalty encoding; Figure 3) and a setting of parametric constraints (Figure 7). Generally, encodings allow us to go beyond the standard SA setting and consider any function that we can train a NN with. This can include nonlinear elements (as in, for example, the Vertex Distance encoding), violations of constraints (for example, Boundary Distance Encoding), or other aspects that are difficult to frame as a LP but easy to encode using a NN. Additionally, we can consider the entire problem space (as long as the NN was successfully trained on this space) and get broader insights than only considering the area around the optimal solution (see Figure 3). Overall, utilizing encodings is promising in settings where not all constraints are easily mathematically formalizable as a LP, and a more general and flexible setting should be considered.

Next to the choice of encoding, the attribution method determines the kind of attribution. For the purpose of this section, we discuss the relationship between the four attribution methods that have been applied in the experimental section and SA. Since FP and LIME are based on perturbations, attributions can be similar to SA. In both cases, small changes of specific values are considered, and the effect of the change in output is observed. Therefore, some encodings would produce similar or even the same kind of insights as SA can provide already. As we show in Section 5.3.1 (Figure 8 in particular), Saliency also does not behave much differently in terms of its attributions, focusing on even more local patterns than FP or LIME. Since its attributions are based on the local gradient, Saliency is the most similar attribution method to concepts from SA, such as shadow prices. Lastly, IG differs substantially from the other attribution methods. Here, two instances are compared (the baseline and the input), and the resulting attribution describes how "responsible" each feature is for the difference in output between baseline and input. This type of attribution is unlike the common procedure applied in SA. Combined with the breadth of choices for different encodings, IG can result in attributions that are inherently different from the type of explanations that SA aims to produce.

In summary, SA provides tools for effectively calculating how small changes of different values impact the optimal solution. While in comparison, our approach requires training a NN, doing so successfully opens up several new opportunities that come with the possibility of choosing different encodings and attribution methods. In addition to our experimental evaluation in this paper, this also means that new XAI methods for NNs that may yet be discovered can use this basic approach and be applied to LP encodings as well, potentially opening up new possibilities in the future. So while there is a motivational overlap between our approach and SA, these approaches are inherently different. In this paper, we do not claim to introduce a "better" variant of SA, but we introduce novel strategies for gaining insights different from what SA offers.

2 SUPPLEMENTARY FIGURES

This section includes the figures referenced in the previous section of the supplementary material.

2.1 Figures

REFERENCES

Bazaraa, M. S., Jarvis, J. J., and Sherali, H. D. (2008). *Linear programming and network flows* (John Wiley & Sons)

Gal, T. (1986). Shadow prices and sensitivity analysis in linear programming under degeneracy: state-of-the-art-survey. *Operations-Research-Spektrum* 8, 59–71

Saltelli, A. and Annoni, P. (2010). How to avoid a perfunctory sensitivity analysis. *Environmental Modelling & Software* 25, 1508–1517

Sokolinsky, L. B. and Sokolinskaya, I. M. (2021). Fragenlp: A generator of random linear programming problems for cluster computing systems. In *International Conference on Parallel Computational Technologies* (Springer)

Ward, J. E. and Wendell, R. E. (1990). Approaches to sensitivity analysis in linear programming. *Annals of Operations Research* 27, 3–38

Zeiler, M. D. (2012). Adadelta: an adaptive learning rate method. *arXiv preprint arXiv:1212.5701*

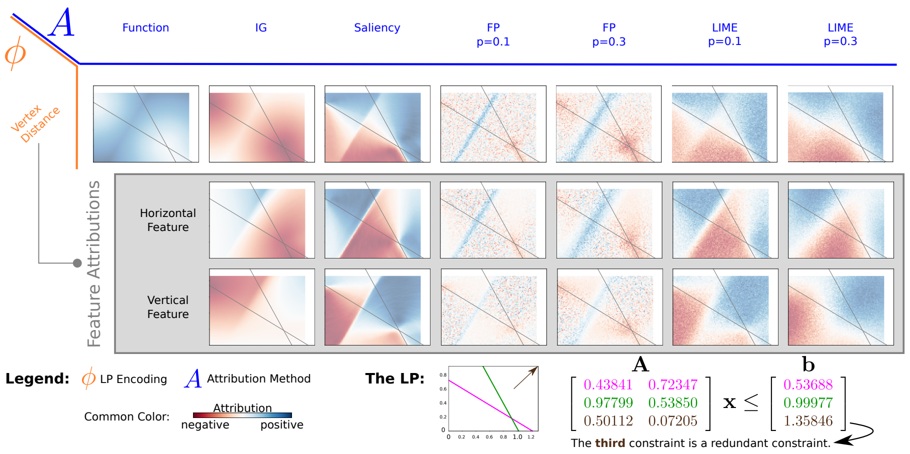

Figure S1. Single feature attributions for the Vertex Distance encoding. The underlying data is the same as for the corresponding figure in the main paper. The LP has two features, one horizontal and one vertical, where the grey lines indicate the constraints for that LP. The line on the top shows the summed-up attribution of all features for the Vertex Distance encoding. Each column shows a different attribution method (or configuration). For FP and LIME, p indicates the maximum possible perturbation in any direction. The (rounded) numbers for the constraints of that LP are shown on the bottom right. The part enclosed in the grey box titled “Feature Attributions” shows attribution for both single features on the Vertex Distance encoding (best viewed in color)

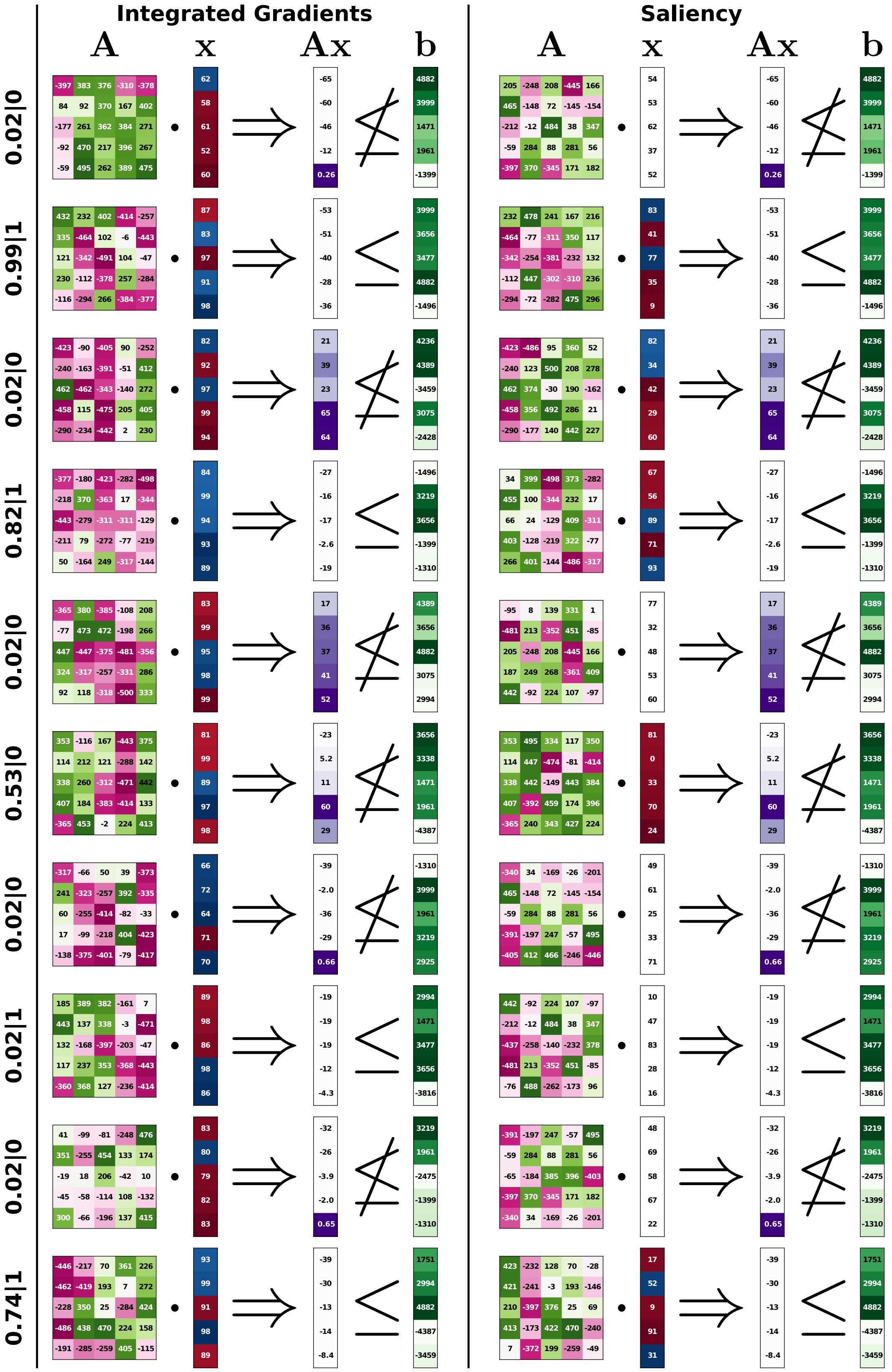

Figure S2. Additional Results for the Large-Scale Experiment. Because of the large dimensional of the LP, only selected columns and rows are shown. $\mathbf{Ax}$ was calculated using the full LP before selecting the depicted rows. The values in $\mathbf{Ax}$ are in the order of 10^4. The colors in $\mathbf{A}$ serve as an additional indicator of the elements (negative values are dark pink/purple, positive values are green). The model prediction and the true class are written on the left side. All values are rounded

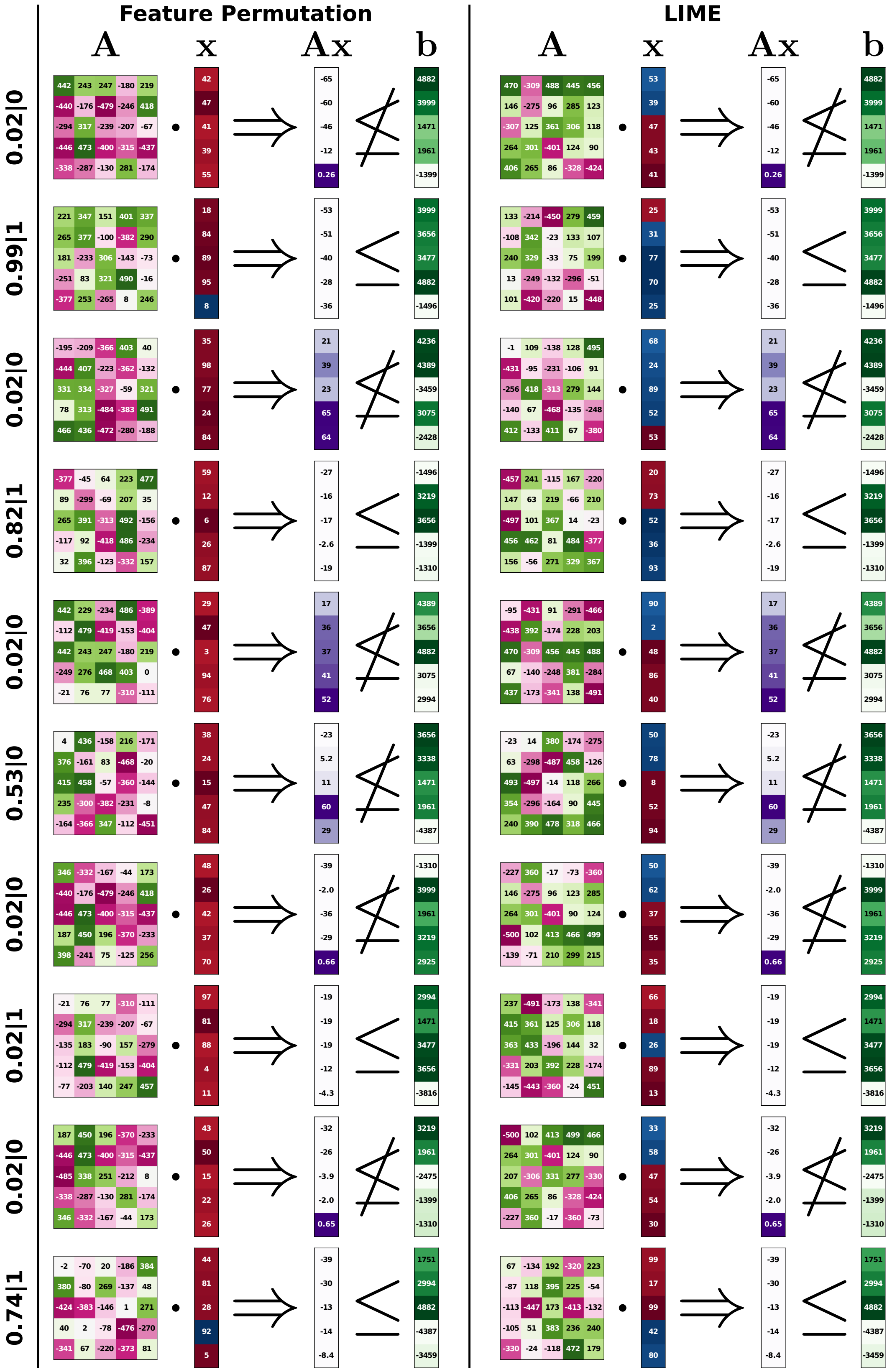

Figure S3. Additional Results for the Large-Scale Experiment. Because of the large dimensional of the LP, only selected columns and rows are shown. $\mathbf{Ax}$ was calculated using the full LP before selecting the depicted rows. The values in $\mathbf{Ax}$ are in the order of 10^4. The colors in $\mathbf{A}$ serve as an additional indicator of the elements (negative values are dark pink/purple, positive values are green). The model prediction and the true class are written on the left side. All values are rounded
